# A Randomized, Open-Label Study to Assess Efficacy of Weekly Assumption of Cholecalciferol versus Calcifediol in Older Patients with Hypovitaminosis D

**DOI:** 10.3390/geriatrics7010013

**Published:** 2022-01-07

**Authors:** Chukwuma Okoye, Valeria Calsolaro, Filippo Niccolai, Alessia Maria Calabrese, Riccardo Franchi, Sara Rogani, Giulia Coppini, Virginia Morelli, Nadia Caraccio, Fabio Monzani

**Affiliations:** Geriatrics Unit, Department of Clinical and Experimental Medicine, University of Pisa, Via Savi 10, 56126 Pisa, Italy; chukwuma.okoye@phd.unipi.it (C.O.); valina82@gmail.com (V.C.); filippo.niccolai@alice.it (F.N.); alessiamariacalabrese@gmail.com (A.M.C.); riccardo.franchi@me.com (R.F.); sara_rogani@hotmail.it (S.R.); giulia.coppini1@gmail.com (G.C.); virginiamorelli93@gmail.com (V.M.); nadiacaraccio.amb@libero.it (N.C.)

**Keywords:** hypovitaminosis D, cholecalciferol, calcifediol, vitamin D, older patient

## Abstract

The aim of this single-center, open-label, randomized controlled study was to evaluate which formulation of vitamin D—between cholecalciferol and calcifediol—is most effective in the treatment of hypovitaminosis D in older adults. Demographic characteristics, clinical history, and comprehensive geriatric assessment were recorded at admission. Eligible patients were randomly assigned an equivalent vitamin D supplement, either with cholecalciferol or calcifediol, from the time of hospital admission to three months after discharge. Among the 140 older patients included (mean age 83 ± 6.6 years, 57.8% females), 69 received cholecalciferol and 71 received calcifediol. The mean plasma values of 25-hydroxyvitamin D3 (25OH-vitamin D3) found at the time of enrollment were 16.8 ± 9.9 ng/mL in patients receiving cholecalciferol and 18.8 ± 13.3 ng/mL in those treated with calcifediol (*p* = 0.31). At the three month follow-up, the mean concentration of 25OH-vitamin D3 was significantly higher in patients treated with calcifediol than in those receiving cholecalciferol (30.7 ± 8.4 vs. 45.4 ± 9.8 ng/mL, respectively; *p* < 0.001). Supplementation with either cholecalciferol or calcifediol effectively results in reaching the optimal circulating values of 25OH-vitamin D3 in older patients suffering from hypovitaminosis D. However, supplementation with calcifediol led to average circulating values of 25OH-vitamin D3 that were significantly higher (over 50%) than those obtained with cholecalciferol.

## 1. Introduction

Hypovitaminosis D represents a widespread condition worldwide, particularly in the elderly population; it is estimated that about 7% of the world population is affected by severe hypovitaminosis ([25-hydroxyvitamin D3 (25OH-vitamin D3)] less than 10–12 ng/mL), while 37% of the population has moderate hypovitaminosis (25OH-vitamin D3 between 20 ng/mL and 10–12 ng/mL) [1]. There is broad consensus in the literature on how achieving sufficient levels of vitamin D plays an important role in improving not only bone homeostasis but also muscle performance and physical health [2,3,4,5]. The therapeutic strategy most often used to reach adequate levels of vitamin D is administering vitamin D supplements with a correct daily intake of calcium, the latter preferably with food [4]. Cholecalciferol (D3) and ergocalciferol (D2) have been used as supplements for a long time. Recently, calcifediol, the form activated by the hepatic enzyme 25-hydroxylase, has also been considered a valid therapeutic alternative [6].

Even though cholecalciferol and calcifediol are related molecules, they present several differences from the pharmacokinetic point of view. The first noticeable difference is represented by the half-life; in particular, the half-life of cholecalciferol is around two months, compared to the two weeks of calcifediol, due to the higher lipophilicity of the former molecule [7]. Thanks to its higher lipophilia, cholecalciferol is accumulated in the adipose tissue and is released gradually over time in a sort of self-regulation, which allows for the intermittent administration of the chosen dose and higher compliance among patients [7]. Calcifediol has a quicker elimination rate; therefore, a sufficient dosage should be administered on a daily or weekly basis [8]. Moreover, there is a different affinity for the vitamin D receptors (VDR), which is lower for cholecalciferol than calcifediol [7,9,10]. The intestinal absorption of cholecalciferol is effective among healthy subjects, while it might be severely compromised in patients with intestinal malabsorption [11]—a condition which does not spare geriatric patients [12,13]. On the contrary, calcifediol is absorbed very effectively and the difference in intestinal absorption kinetics largely explains its remarkable bioavailability [6]. 

Few studies have evaluated differences in the efficacy of the two compounds in correcting vitamin D deficiency among different age groups—mainly young adults and post-menopausal women [14,15,16,17,18]; however, data in the older and oldest-old population are very scarce, and only Ruggiero et al. considered a population with a mean age >80 years [19]. Given the paucity of data for individuals in this specific age range, who are often hospitalized for fragility fractures and would benefit most from vitamin D supplementation, we conducted a prospective, randomized study to evaluate which vitamin D formulation—between cholecalciferol and calcifediol—is the most effective in treating hypovitaminosis D in older adults.

## 2. Materials and Methods

A single-center, open-label, randomized controlled study was conducted enrolling geriatric patients consecutively hospitalized in the Geriatric Unit of the University Hospital of Pisa for acute illness from May to September 2020. No age restriction was applied, and we enrolled patients with 25OH-vitamin D3 levels < 30 ng/mL. Demographic characteristics and clinical history were collected at the time of admission. Within the first 24 h of admission, each patient underwent a comprehensive geriatric assessment (CGA), composed of the following measures: Cumulative Illness Rating Scale (CIRS) [20], Activities of Daily Living (ADL) [21], Instrumental Activities of Daily Living (IADL) [22], Short Portable Mental Status Questionnaire (SPMSQ) [23], Mini-Nutritional Assessment (MNA) [24], and Exton Smith Scale (ESS) [25]. Individuals’ body mass index (BMI) and multi-prognostic index (MPI) [26] values were also recorded. In order to investigate the presence of sarcopenia, the handgrip strength (HGS) test was performed on the dominant hand [27] using a hand dynamometer. Participants were seated with their shoulder adducted, elbow flexed to 90 degrees, and forearm and wrist neutral. The highest score out of three consecutive measurements was recorded. The study exclusion criteria were: i, having received vitamin D supplementation during the past six months; ii, stage V renal insufficiency; iii, liver failure (defined as a Child–Pugh classification of a B or C); iv, hyperparathyroidism; v, malabsorption syndromes or the long-term prescription of drugs reducing vitamin D absorption (i.e., antiepileptic drugs, long-term corticosteroids, or bisphosphonates); vi, neoplastic disease under treatment; vii, patients being unable to give informed consent. The dose of vitamin D supplementation was chosen based on current recommendations (20 mcg = 800 UI/day) [28,29,30,31,32]. Considering that calcifediol is about 3-fold more potent than cholecalciferol [10], eligible patients randomly received a bioequivalent dose of vitamin D—either with cholecalciferol (10,000 IU/mL, equivalent to 70 drops/week, 437.5 mcg/week) or calcifediol (1.5 mg/10 mL equivalent to 28 drops/week, 140 mcg/week)—once each week on the same day and at the same time (after lunch) during hospitalization and for three months after discharge. Randomization was performed by a physician using coin-flipping procedure. Before starting vitamin D supplementation, baseline blood samples were taken the first morning after admission, after an overnight fast, at 6 a.m.; 25OH-vitamin D3, parathyroid hormone, total calcium, calcium ion, phosphate, albumin, and creatinine were measured. 25OH-vitamin D3 levels were measured (blood samples were collected at the baseline evaluation, and the relative plasmas were stored at −20 °C) by tandem mass spectrometry coupled with high performances liquid chromatography (HPLC-MS-MS), using the MSMS VitD Kit from PerkinElmer (Waltham, MA, USA). A standard biochemical blood sample analysis was performed by Roche Autoanalyzer (Indianapolis, IN, USA) at the central laboratory of the University Hospital of Pisa. Three months after discharge, patients were re-evaluated at the geriatric-endocrinology outpatient clinic, where they underwent an HGS test and blood tests. The study protocol complied with the Declaration of Helsinki and was approved by the Pisa University Hospital Ethics Committee (n° protocol: CEAVNO-881/2020). Written informed consent was obtained from each enrolled patient.

### Statistical Analysis

SPSS statistical software (IBM SPSS version 27.0, IBM Corporation, Chicago, IL, USA and its licensor 1989–2020) was utilized for the entire statistical analysis, whereas GraphPad Prism 9 was utilized for the graph plotting. A sample size of 58 for each group in the study achieved 90% power to detect a 15% difference among the means versus the alternative of equal means using an F test at a 0.05 significance level. The size of the variation in the means is represented by 0.25 of their standard deviation. The results were analyzed for normal distributions using a Shapiro–Wilk test, while homogeneity was tested using Levene’s test. Vitamin D levels were submitted to explanatory analysis for both a normal distribution and homoscedasticity. Continuous variables were presented as means ± standard deviations, ordinal variables were presented as medians and interquartile ranges (IQRs), and categorical variables were presented as percentages. Mann–Whitney and chi-square tests were used for multiple comparisons. Two-factor ANOVAs with repeated measures for time and the adjustment of *p* values using the Greenhouse–Geisser epsilon were performed in order to evaluate mean differences for the between-subjects model (factors: time and group) and the within-subjects model (factors: time and time for each group) among patients receiving vitamin D supplementation and their counterparts during the follow-up. Tests were performed considering a level of significance of 5%. 

## 3. Results

**Figure 1:**
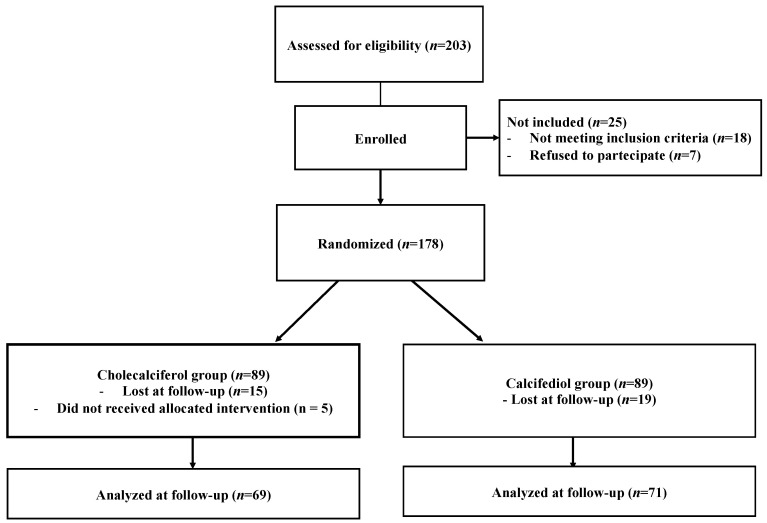
Flowchart of study enrollment.

Overall, 140 patients were included in the study (Figure 1), 69 received cholecalciferol (56.5% women, mean age 84.9 ± 6.4 years), and 71 received calcifediol (59.1% women, mean age 82.7 ± 6.7 years). As reported in Table 1, the two groups did not differ in terms of the reason for admission, comorbidities, the degree of disability [ADL median (IQR): 5(2) vs. 6(1), *p* = 0.42; IADL median (IQR): 4(5) vs. 5(4), *p* = 0.42], nutritional status [BMI median (IQR): 23.7(7.2) vs. 25(5.6), *p* = 0.95, MNA median (IQR): 23(8) vs. 25(6), *p* = 0.55], or strength as estimated through the HGS test (mean 17.5 ± 7.2 vs. 17.3 ± 7.2, *p* = 0.92). No statistical differences were found between the HG test and 25OH-vitamin D3 levels using the Spearman’s correlation analysis (Spearman’s rho =0.50, *p* = 0.30). Moreover, patients showed a similar degree of frailty as expressed using the MPI (mean 0.39 ± 0.20 vs. 0.32 ± 0.18, *p* = 0.37). Main acute illnesses requiring hospitalization were as follows: heart failure (10.7%: 10.1% in the cholecalciferol and 11.3% in the calcifediol group), arrhythmia (3.6%: 2.8% in the cholecalciferol and 4.2% in the calcifediol group), acute respiratory failure (12.9%: 13.0% in the cholecalciferol and 12.7% in the calcifediol group), bleeding (6.4%, 7.2% in the cholecalciferol and 5.6% in the calcifediol group), acute kidney failure (2.9%: 1.5% in the cholecalciferol and 4.2% in the calcifediol group), electrolyte disorders (2.9%: 2.9% in the cholecalciferol and 2.8% in the calcifediol group), stroke (7.9%: 7.2% in the cholecalciferol and 8.4% in the calcifediol group), decompensated diabetes (3.6%: 2.9% in the cholecalciferol and 4.2% in the calcifediol group), sepsis (10.7%: 11.5% in the cholecalciferol and 9.8% in the calcifediol group), and miscellaneous illnesses (38.6%: 37.6% in the cholecalciferol and 39.4% in the calcifediol group), with no significant differences between the two groups. 

**Figure 2:**
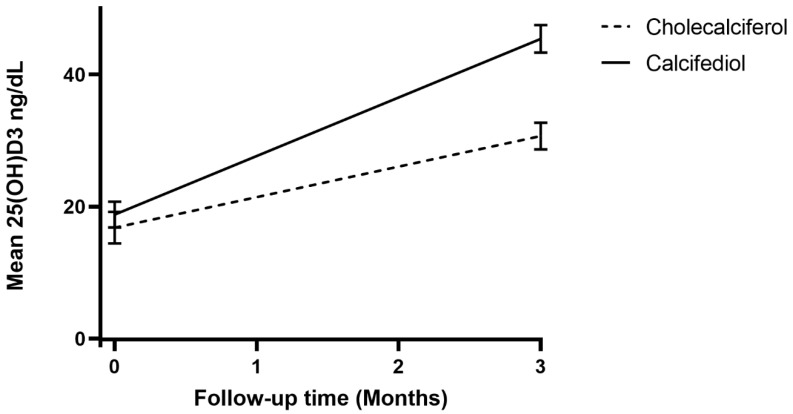
Mean 25OH-vitamin D3 values over time, according to the type of vitamin D supplementation.

In regard to biochemistry blood exams, no significant differences were found in terms of serum creatinine concentration (1.15 ± 0.92 vs. 1.21 ± 1.02 mg/dL, *p* = 0.24), PTH circulating levels (48.1 ± 39.6 vs. 60.7 ± 36.9 pg/mL, *p* = 0.17), calcium concentration (8.8 ± 0.4 vs. 9 ± 0.4 mg/dL, *p* = 0.052), phosphoremia (3.2 ± 0.5 vs. 3.3 ± 0.8 mg/dL, *p* = 0.35), or albumin concentration (3.5 ± 0.4 vs. 3.5 ± 0.4 g/dL, *p* = 0.64). The mean plasma values of 25OH-vitamin D3 found during enrollment were 16.8 ± 9.9 ng/mL in patients receiving cholecalciferol and 18.8 ± 13.3 ng/mL in those treated with calcifediol (*p* = 0.31). At the three-month follow-up, the mean concentration of 25OH-vitamin D3 was significantly higher among patients treated with calcifediol than among those receiving cholecalciferol (30.7 ± 8.4 vs. 45.4 ± 9.8 ng/mL, respectively; *p* < 0.0001) (Figure 2).

## 4. Discussion

In the present study, we found that weekly supplementation with calcifediol appears to be more effective compared to a bioequivalent dosage of cholecalciferol in a cohort of older adults. Several studies showed that calcifediol is faster and more potent than cholecalciferol in increasing plasma 25OH-vitamin D3 levels [8,14,15,17,18,19,33,34], although most of these trials excluded the oldest-old population. 

The goal of the prevention and correction of hypovitaminosis D is to achieve serum levels of 25OH-vitamin D3 ≥ 30 ng/mL (75 nmol/L), as recommended by most scientific societies [1]. The main component of the daily requirement of vitamin D derives from the endogenous synthesis in the skin following sun exposure to UVB rays. However, the latter process becomes ineffective with increasing age. The supplementation of vitamin D is the recommended therapeutic strategy, along with sufficient calcium intake [10]. Yet, hypovitaminosis D is frequent in the older and oldest-old (>85) populations [1], and reduced vitamin D levels are linked to greater vulnerability and frailty [3]. As a fact, 25OH-vitamin D3 can regulate the inflammatory response, promoting cyclin-dependent kinase (CDK) inhibitor synthesis, influencing several growth factors, and leading to the containment of systemic inflammation [35,36,37]. In a condition of 25OH-vitamin D3 deficiency, the low calcium concentration induces an increase in circulating PTH, which, through considerable renal reabsorption, increases 1,25OHD production and interaction with RANKL, restoring serum calcium values [38,39,40]. One of the strengths of the current study is that the mean age of patients was significantly higher compared to previous reports [8,14,15,17,18,33,34]; furthermore, we investigated functional status, reporting a high degree of autonomy in ADL in both groups. At baseline, no differences between the two cohorts were found in terms of BMI or MNA, confirming the homogeneity of our sample, similar to that of a previous report on a cohort of oldest-old patients [19]. In our study, although not reaching statistical significance, the finding of higher 25OH-vitamin D3 levels alongside the higher handgrip test values could support the relation between 25OH-vitamin D3 and muscle function [41,42,43]. 

At the 3-month follow-up, both cholecalciferol and calcifediol supplementation resulted in effectively accomplishing the 30 ng/mL threshold of patients’ 25OH-vitamin D3 values. The mean concentration of 25OH-vitamin D3 was significantly higher among patients treated with calcifediol than among those receiving cholecalciferol, further strengthening previous literature data [8,10,14,15,17,18,19,33,34]. These findings can be explained by the different intestinal absorption kinetics in older patients. Indeed, cholecalciferol is transported by chylomicrons and reaches the bloodstream via lymphatic circulation [44,45], while calcifediol is absorbed more effectively (almost 100%) [16], as it is transported directly into the bloodstream via the portal vein [46]. Furthermore, since calcifediol does not require hepatic conversion, it shows a linear relationship between the dose administered and the achieved serum levels [18]. Therefore, considering that elderly patients may experience intestinal malabsorption due to poly-therapies, gut dysbiosis caused by drugs interaction or the pathophysiological aging of the gastrointestinal tract [11,12,47], calcifediol could be more effective in reaching optimal vitamin D levels. In conclusion, the present study confirms previous findings from Ruggiero et al., but includes a larger cohort [19] and provides additional evidence regarding the oldest-old population, which is usually under-represented in clinical trials. Compared to previous findings [16,19], 25OH-vitamin D3 levels in our cohorts are higher at baseline as well as at the three-month follow-up; a possible explanation could be that we excluded all patients with malabsorption and those taking medications that could reduce vitamin D absorption.

Nonetheless, our study has some limitations. We acknowledge that the study schedule of a three-month follow-up visit is rather long; however, the enrollment window was during the COVID-19 pandemic; therefore, we tried to avoid patients’ and caregivers’ access to laboratories or outpatient clinics for as much as possible. Participants administered both of the vitamin D supplementations at home, in absence of an investigator confirmation; however, also according to hospital policy during the pandemic, we assessed patients’ adherence to the therapy as well as the possible onset of acute events via phone assessment on a monthly basis. Finally, results from our study are superimposable with previous reports carried out in similar cohorts of older patients, which underlines the reliability of our findings. 

## 5. Conclusions

This study documents how three months of either cholecalciferol or calcifediol supplementation effectively results in reaching the optimal circulating values of 25OH-vitamin D3 in older patients suffering from hypovitaminosis D. However, supplementation with calcifediol shows average circulating values of 25OH-vitamin D3 to be significantly higher (over 50%) than those obtained with cholecalciferol. Further multi-center studies are nonetheless needed to confirm these findings.

## Figures and Tables

**Table 1:** Clinical characteristics of the study population.

	All Patients *N* = 140	Cholecalciferol *N* = 69	Calcifediol *N* = 71	*p*-Value
Female (%)	81 (57.8)	39 (56.5)	42 (59.1)	0.75
Age (years, mean, SD)	83.8 (6.6)	84.9 (6.4)	82.7 (6.7)	0.052
BMI (median, IQR)	24.4 (6.1)	23.7 (7.2)	25 (5.6)	0.95
ADL (median, IQR)	6 (2)	5 (2)	6 (1)	0.42
IADL (median, IQR)	4 (5)	4 (5)	5 (4)	0.42
MNA (median, IQR)	25 (5)	23 (8)	25 (6)	0.55
Exton Smith Scale (median, IQR)	18 (3)	17 (3)	18 (3)	0.74
SPMSQ (median, IQR)	2 (2)	2 (2)	2 (3)	0.68
CIRS–C (median, IQR)	3 (2)	3 (2)	3 (3)	0.37
MPI (mean, SD)	0.35 (0.19)	0.39 (0.20)	0.32 (0.18)	0.37
Arterial hypertension (%)	98 (70)	48 (69.6)	50 (70.4)	0.33
CAD (%)	15 (10)	8 (11.6)	7 (9.9)	0.32
AF (%)	26 (18.6)	12 (17.4)	14 (19.7)	0.77
Heart failure (%)	57 (40.7)	29 (42)	28 (39.4)	0.57
Diabetes (%) CKD (%)	29 (20.7) 32 (22.8)	14 (20.3) 16 (23.2)	15 (21.1) 16 (22.5)	0.42 0.30
COPD (%)	13 (9.3)	6 (8.6)	7 (9.8)	0.54
Number of drugs (median, IQR)	6 (3)	7 (3)	6 (3)	0.65
Creatinine mg/dl (mean, SD)	1.15 (0.53)	1.15 (0.92)	1.21 (1.02)	0.24
PTH ng/dL (mean, SD)	55.3 (38.3)	48.1 (39.6)	60.7 (36.9)	0.17
Serum Calcium mg/dl (mean, SD)	8.9 (0.4)	8.8 (0.4)	9.0 (0.4)	0.052
Serum Phosphate mg/dl (mean, SD)	3.25 (0.8)	3.2 (0.5)	3.3 (0.8)	0.35
Serum Albumin g/dl (mean, SD)	3.5 (0.4)	3.5 (0.4)	3.5 (0.4)	0.64
Handgrip test (mean, SD) Males Females	17.4 (7.4) 25.9 (5.7) 13.9 (4.8)	17.5 (7.2) 24.3 (5.4) 13.7 (4.4)	17.3 (7.2) 27.1 (6,2) 14.1 (5.3)	0.92 0.34 0.82
25OHVitD at study enrollment (ng/mL)	17.8 (11.7)	16.8 (9.9)	18.8 (13.3)	0.31
25OHVitD at 3-month follow-up (ng/mL)	38.1 (18.3)	30.7 (8.4)	45.4 (9.8)	<0.001
25OHVitD3 mean difference at 3 months (SEM)	20.2 (+17.8; +23.2)	13.7 (+11.8; +15.3)	26.6 (+22.9; +30.1)	<0.001

BMI, body mass index; ADL, Activities of Daily Living; IADL, Instrumental Activities of Daily Living; MNA, Mini-Nutritional Assessment; SPMSQ, Short Portable Mental Status Questionnaire; CIRS-C, Cumulative Illness Rating Scale-Comorbidity; MPI, Multi Prognostic Index; PTH, parathyroid hormone; CAD. coronary heart disease; AF, atrial fibrillation; CKD: chronic kidney disease; COPD: chronic obstructive pulmonary disease.

## Data Availability

The data presented in this study are available upon request from the corresponding author.
